# Adverse reactions to food: Navigating the maze in primary health care

**DOI:** 10.4102/safp.v64i1.5530

**Published:** 2022-07-21

**Authors:** Jurgens Staats, Idonette Van Zyl

**Affiliations:** 1 Department of Family Medicine, Faculty of Health Sciences, University of the Witwatersrand, Johannesburg, South Africa; 2 Department of Nutrition, Faculty of Health Sciences, North-West University, Potchefstroom, South Africa

**Keywords:** food allergy, food hypersensitivity, food intolerance, allergy-focused history, allergy diagnosis

## Abstract

Primary health care providers are often confronted with patients describing adverse reactions to food. With a multiplex of differential diagnoses, it is essential to have a structured approach to these complaints. Just as allergies can have a negative impact on quality of life, inappropriate food allergy labelling can cause unnecessary distress and hardship for patients. Understanding the diagnostic approaches to adverse food reactions, alongside an appreciation of multidisciplinary collaboration, can assist the clinician in making appropriate decisions in the management of these patients.

## Introduction

**FIGURE 1:**
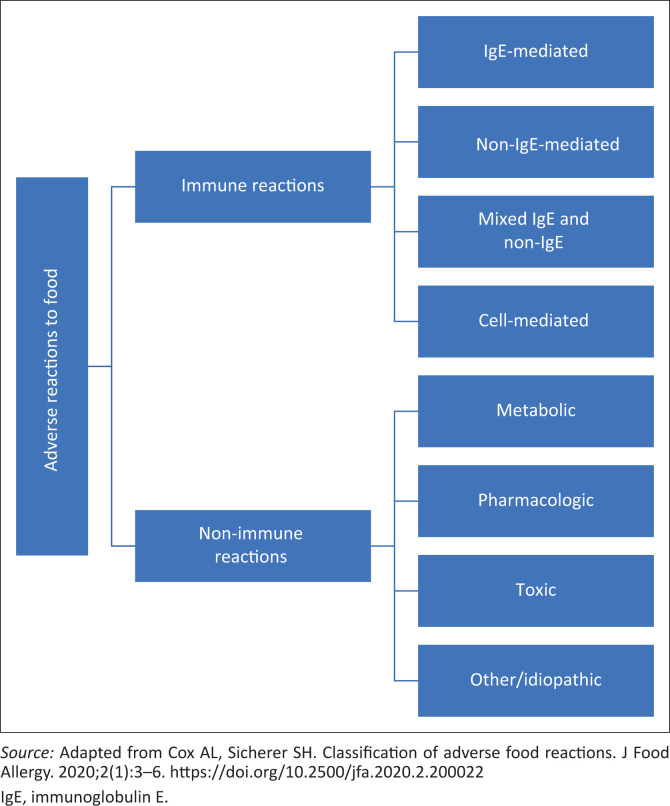
Classification of adverse food reactions.

An adverse food reaction is defined as an abnormal response linked to the ingestion of food and can be broadly classified into immune-modulated reactions and non-immune-modulated reactions (food intolerances) as demonstrated in Figure 1.^1^ It is a common complaint, affecting 15% – 20% of the global population, with multisystem involvement, ranging from gut to mucocutaneous, respiratory, cardiovascular and central nervous system symptoms.^2^ In addition, patients and caregivers may suffer from anxiety and reduced quality of life related to attending or being excluded from social events, bullying at school, the affordability of special or ‘free-from’ products and the extra time spent in-store to read food product labels. Fear of recurrence after events such as anaphylaxis may also lead to conditions such as post-traumatic stress reactions.^3^

Whilst the majority of adverse reactions to food can be attributed to non-allergic processes,^4^ patients may label their reactions as food allergy (FA). This self-reported diagnosis may lead to an overestimation as much as four times higher than the true prevalence,^5^ which may lead to self-imposed food avoidance^6^ and a risk of nutritional deficiencies.^7^

Food allergy is an adverse food reaction associated with a reproducible hypersensitive immune response.^6^ Despite beliefs that FAs are rare in Africa, evidence suggests that it is a growing problem^8^ and that it remains underdiagnosed.^5^ Studies have found that the true prevalence of immunoglobulin E (IgE)-mediated FA in children in South Africa varies from 0.5% in rural populations to 2.5% in urban populations,^9^ whilst the prevalence of IgE sensitisation to common foods is estimated at 11.6%.^10^ At present, non-IgE-mediated FAs (non-IgE FA) are less well studied than other food allergies.^11^ There is currently no prevalence data available for non-IgE FA in South Africa.

It is the dichotomy of the increasing incidence of FA against the backdrop of self-reported overestimation that creates a diagnostic conundrum for primary health care providers. Considering that there are no validated tests for non-IgE-mediated allergies yet, clinicians may find themselves stuck in a maze of uncertainty.

The clinician should be able to discern a true FA from food intolerance and therefore provide relevant counselling or make an appropriate referral. Truly allergic individuals are at risk of developing life-threatening anaphylaxis and should therefore receive education on avoidance measures as well as the need for an allergy action plan and adrenaline autoinjector. Conversely, those without concrete evidence of harmful effects because of food ingestion should be discouraged from practising unnecessary avoidance.

It is therefore essential to have a structured approach to the diagnosis of an FA.

## Diagnostic approach to food allergies

The diagnosis of FA may be seen as a process of triangulation involving sequential steps. One such model recommended by the European Academy of Allergy and Clinical Immunology supports a five-step approach that can be followed to confirm or exclude an FA^7^:

- Food allergy-focused history
- Selecting appropriate investigations to determine the presence of sensitisation
- Short-term elimination of suspected foods for diagnostic purposes
- Performing oral food challenges
- Evaluating for non-IgE-mediated FA.

### Step 1: Food allergy-focused history taking

An allergy-focused clinical history remains the cornerstone of any investigative process. Ignoring this step in favour of other uninformed investigations will likely create further diagnostic confusion. The EATERS (exposure, allegen, timing, environment, reproducible, symptoms) mnemonic^2^ provides a method for clinicians to interpret each case and makes sense of test results (Table 1).

**TABLE 1:** EATERS mnemonic.

Focus	IgE-mediated allergies	Non-IgE-mediated allergies
Exposure	Proximity to allergens is not deemed adequate to suggest exposure, the suspect food must be ingested. Other occasional methods for exposure could include handling raw food, being kissed by someone who has just eaten or inhalation of aerosolised pan-fried foods.	Ingestion of allergens via infant formula, food consumption or exposure through allergens excreted in breastmilk.^12^
Allergen	The most common allergens include milk, egg, nuts, soy, wheat and fish. The allergen is often determined by host immune responses.	Any food can cause a reaction. Cow’s milk, soy and egg are often implicated. More common foods include corn, wheat, rice, oat, barley, sweet potato, white potato, chicken, vegetables, fruit, peanuts, nuts, fish and shellfish.^7,12^
Timing	Most reactions occur immediately on exposure.	Symptoms are often delayed and typically occur within 72 h after ingestion.
Environment	Weaning is the most common scenario as new foods are introduced to the diet. Food allergy also tends to occur when eating away from home – nurseries, restaurants, parties and holidays.	More commonly seen in young infants and children. May occur during breastfeeding, formula feeding or during the introduction of solid foods.
Reproducible	Strictly reproducible and occur at all subsequent exposures to the food.	Strictly reproducible, and symptoms may occur with small volumes of the implicated food.
Symptoms	*Skin*: hives, swelling, redness, itching	*Skin*: pruritus, erythema, atopic eczema
	*GIT*: vomiting, abdominal pain, diarrhoea	*GIT*: acute or chronic diarrhoea,^13^ gastroesophageal reflux, loose or frequent stools, constipation, food aversion or refusal, abdominal pain, severe or recurrent vomiting, intermittent bloody stools^11^ or mucous in stools, dysphagia, oesophageal food impaction, erythema around the anus, malabsorption
	*Respiratory tract*: chough, wheeze, stridor, hoarseness, hypoxaemia *Cardiovascular*: shock, hypotension, syncope, collapse	*Others*: failure to thrive, changed behaviour because of discomfort and pain, persistent clear rhinorrhoea, lethargy, possible cyanosis, repetitive emesis may be associated with progressive lethargy, shock, dehydration and acidosis, hypotonia and hypotension.^14^

*Source*: Adapted from Erlewyn-Lajeunesse M, Weir T, Brown L, et al. Fifteen-minute consultation: The EATERS method for the diagnosis of food allergies. Arch Dis Child Educ Pract. 2019;104(6):286–291. https://doi.org/10.1136/archdischild-2018-316397

Some caveats may not fit in with the classical history of FA. For instance, food-dependent, exercise-induced anaphylaxis occurs when food and exercise are tolerated independently, but reactions occur when physical exertion follows ingestion of a food allergen.^15^ Secondly, a condition called alpha-gal syndrome presents a true IgE-mediated allergy to red meat, but the timing of symptoms is delayed by 2–6 h,^16^ resulting in the phrase ‘midnight anaphylaxis’.^17^ These examples provide the potential limitations of an allergy-focused history and further demonstrate the fact that diagnosis in allergic conditions is a process rather than a single investigation.

### Step 2: Investigations to confirm sensitisation

Immunoglobulin E (IgE) sensitisation can be determined via skin prick testing (SPT) or specific IgE levels. It is important to remember that sensitisation may be used as a proxy for confirming FA in the context of a positive history.^4^ Food-specific SPT and IgE have a sensitivity of 70% – 100%, but a specificity of 40% – 70%. Therefore, in isolation has limited clinical value as it is possible for patients to be ‘sensitised’, but not clinically allergic to certain foods.^18^ For some allergens, certain threshold levels have been determined to provide a 95% positive predictive value decision point. Notably, the size of the SPT reaction or the level of IgE correlates only to diagnostic probability and not the severity of reactions.^19^

Food-specific immunoglobulin G (IgG) testing is increasingly used by primary health care providers to identify and diagnose food sensitivities. This practice has no evidence basis. Conversely, the development of IgG or IgG4 is used as a marker of desensitisation and/or tolerance to that specific food allergen. Similarly, other tests such as the antigen leukocyte antibody test, provocation–neutralisation testing, hair analysis, electrodermal testing and applied kinesiology are frequently used to detect FA but have not been validated. These testing methodologies require further research for validation, regulation and standardisation before they can be endorsed for clinical practice.^20^

### Step 3: Short-term elimination for diagnostic purposes

Short-term elimination or exclusion diets may be considered in situations where patients suffer from chronic symptoms or when a high index of suspicion exists for a specific food. If the patient improves following the period of elimination, a rechallenge with the suspected foods is done one by one to confirm the presence of a true FA. Low-dose re-introduction is usually done after 2–6 weeks of strict elimination. In instances where no improvement is observed, the eliminated food is unlikely to be the cause of the symptoms, and the value of excluding it from the diet is limited.^21^

### Step 4: Oral food challenges

An oral food challenge is the gold standard for the diagnosis of FA. However, it is mostly reserved for scenarios where further clarification is required (e.g. in the setting of a suggestive history but equivocal SPT or IgE results),^21^ as it is time-consuming and carries a risk of inducing a severe allergic reaction. It should therefore be done under the supervision of a trained clinician.^22^

### Step 5: Evaluation for non-IgE food allergy

#### Overview of non-IgE-mediated food allergy

Non-IgE FAs are characterised by gastrointestinal reactions, including food protein-induced allergic proctocolitis (FPIAP), food protein-induced enterocolitis syndrome (FPIES) and food protein-induced enteropathy (FPE) (see Box 1 for disease specific characteristics); cutaneous reactions (such as atopic dermatitis, contact dermatitis and dermatitis herpetiformis) and respiratory reactions (such as Heiner syndrome) are often reported. Eosinophilic gastrointestinal disorders (EGIDs), including eosinophilic esophagitis (EoE), allergic eosinophilic gastroenteritis (AEG) and eosinophilic colitis (EC), are also classified as non-IgE FAs. Eosinophilic gastrointestinal disorders often present with non-specific symptoms such as repeated regurgitation, vomiting, and watery, mucoid or haemorrhagic diarrhoea, combined with other features such as poor growth and crying crises (colic). Food allergy itself can also cause gastroesophageal reflux disease.^23,24^

BOX 1 Characteristics of disease-specific conditions.

- FPIAP symptoms are induced by localised inflammation of the distal colon, causing blood/mucus-streaked stools, together with mild diarrhoea in otherwise well-appearing infants.
- FPE mainly affects the small intestine, resulting in symptoms including intermittent vomiting, diarrhoea and malabsorption with failure to thrive.
- FPIES can affect the entire gastrointestinal tract, presenting with intractable vomiting, often followed by diarrhoea, which can result in metabolic disturbances and hypovolemic shock. FPIES can further be classified according to the timing of symptoms (acute vs. chronic FPIES), the severity of clinical manifestations (mild, moderate and severe), age of onset (early-onset, late-onset, adult FPIES), type of food triggers (cow’s milk/soy vs. solid foods) and the presence of food-specific IgE (sIgE) (atypical FPIES).^28^

*Source:* Labrosse R, Graham F, Caubet J-C. Non-IgE-mediated gastrointestinal food allergies in children: An update. Nutrients. 2020;12(7):2086. https://doi.org/10.3390/nu12072086

FPIAP, food protein-induced allergic proctocolitis; FPE, food protein-induced enteropathy; FPIES, food food protein-induced enterocolitis syndrome.

Non-IgE-mediated food allergic gastrointestinal disorders, including gastroesophageal reflux, abdominal pain, constipation and frequent vomiting, can adversely affect a child’s feeding ability and skills, depending on the associated symptoms, disease severity, age at which the child is affected, social circumstances and the degree of dietary restriction recommended. Early diagnosis and management of these feeding difficulties are of utmost importance, as they can result in nutritional deficiencies or growth faltering.^25^ Growth faltering is a commonly described symptom, with low weight and in particular low height found in approximately 10% of children with non-IgE FA.^26^ Cow’s milk is a predominant FA in children, of which non-IgE gastrointestinal FA accounts for up to 50% of reactions. Similarly, cow’s milk is responsible for the majority of reactions in FPIES, FPE and FPIAP.^28^

#### Diagnostic approach to non-IgE-mediated food allergy

With the lack of laboratory tests to support the diagnosis of non-IgE FAs, the diagnosis remains challenging, as it relies on clinical evaluation and sound history taking (refer to Table 1). The onset of non-IgE FA symptoms is delayed and may have a chronic presentation, making their association with the food allergen less evident.^27^ The diagnosis of non-IgE FAs is based on the occurrence of symptoms after the culprit allergen is consumed, followed by their resolution once the suspected food has been eliminated and reappearing when it is re-introduced.^28^

## Multidisciplinary collaboration: The role of the registered dietitian

A targeted, individualised approach to each allergic patient remains essential for the effective management of FA. As part of a thorough nutritional assessment, an experienced dietitian will conduct an allergy-focused clinical history and a comprehensive dietary assessment, including the evaluation of the food and symptom diary, as well as monitor growth. Dietary management includes advice regarding allergen avoidance, food label ingredients and terminology, hidden allergens in foods, suitable replacement foods and ensuring dietary adequacy. Ideally, no food should be removed from an individual’s diet without the involvement of a dietitian.^29,30^ A dietitian can help with the identification of possible allergen–symptom associations and offer the necessary support to improve the allergic individual’s quality of life.

## Summary

Adverse reactions to food are commonly seen in primary health care providers. In addition to the obvious physical effects, it also has negative psychological and social consequences. Given the variety of causes and presentations, clinicians may easily be confused, resulting in haphazard testing and counselling.

Central to the accurate diagnosis of an FA is a sound allergy-focused clinical history. Specific information regarding the presenting symptoms should include the following:^12,29^ the age of symptom onset; the timing of onset following food contact; the duration, severity and frequency of symptoms; and the quantity of food required to induce a reaction, as is the reproducibility of symptoms on re-exposure. Other pertinent information includes personal history of atopic disease (other FA, asthma, eczema or allergic rhinitis); family history of atopic disease in parents or siblings; cultural and religious factors that affect food preferences; comprehensive dietary assessment, including the age of weaning and food preferences; and whether breastfed, mixed or formula-fed. If the patient is breastfed, enquire about maternal dietary intake; details of any previous treatment, including medication such as antihistamines; and details on food elimination and reintroduction which may guide further investigation and management. A ‘14-day food and symptom diary’ is a valuable diagnostic aide.
